# Impact of pharmacist-led educational services in promoting breast cancer awareness

**DOI:** 10.1186/s12905-025-04035-0

**Published:** 2025-09-29

**Authors:** Nazish Mehmood, Amjad Khan, Sameen Abbas, Saima Mushtaq, Yu Fang, Muhammad Ans, Fakhar-ud-Din, Gul Majid Khan

**Affiliations:** 1 Department of Pharmacy, Quaid-i-Azam University, Islamabad, Pakistan; 2 Department of Pharmacy, The First Affiliated Hospital, Xi’an Jiaotong University, Xi’an, China; 3 Department of Pharmacy Administration and Clinical Pharmacy, School of Pharmacy, Health Science center, Xi’an Jiaotong University, Xi’an, China; 4 Punjab University College of Pharmacy, University of the Punjab, Allama Iqbal Campus, Lahore, Pakistan

**Keywords:** Pharmacist, Breast cancer, Lack of awareness, Breast self-examination, Pakistan

## Abstract

**Background:**

Breast cancer is rapidly increasing worldwide. Pakistan has a high incidence rate of one in every nine women. The lack of awareness is the major reason for delayed diagnosis, thus resulting in high mortality. This study aimed to assess the impact of pharmacist-led breast cancer education at community pharmacies on promoting breast cancer awareness.

**Methodology:**

A longitudinal pre-post intervention study was conducted on 319 participants using a self-designed questionnaire. During the pre-intervention phase, data were collected from participants at various community pharmacies employing questionnaire administration. The provision of a breast cancer educational session followed the session. After three months, the participants were contacted via telephone, and the questionnaires were filled in again during the post-intervention phase. Data was analyzed using SPSS version 25.

**Results:**

The pre-post phase data evaluation reported improved breast cancer awareness among the study participants, with a significant increase (*p* = 0.000) in awareness of breast cancer symptoms, risk factors, and diagnostic techniques. There was a statistical increase in breast self-examination practice during the post-intervention phase (2.5% to 93.8%). Knowledge about clinical breast examination improved from 8.5% (pre-intervention phase) to 84.4% (post-intervention phase). Additionally, understanding mammography as a vital screening technique improved significantly, with approximately 34.7% of eligible women having undergone mammography in the post-intervention phase.

**Conclusion:**

A notable improvement in breast cancer awareness and self-examination was observed through pharmacist-led education. Leveraging pharmacist-led services in community pharmacies could effectively contribute to breast cancer control efforts. Utilizing pharmacists nationwide could facilitate the implementation of comprehensive strategies to elevate public breast cancer awareness nationally.

**Supplementary Information:**

The online version contains supplementary material available at 10.1186/s12905-025-04035-0.

## Introduction

Cancer is a major global public health issue and remains one of the leading causes of mortality worldwide [1]. Among the various types of cancer, breast cancer is the most commonly diagnosed malignancy in women [2, 3]. According to the Global Cancer Observatory (GLOBOCAN) 2020 report, breast cancer accounts for the highest incidence of all cancers, with a global incidence rate of 47.8 per 100,000 women [4]. It also ranks as the second most common cause of cancer-related deaths among women, both in developed and developing nations [1, 5]. The increasing incidence of breast cancer is largely attributed to longer life expectancy, lifestyle changes, and urbanization trends throughout the globe [2].

Early detection of breast cancer significantly improves treatment outcomes and survival rates. The commonly used screening methods for early detection of breast cancer include mammography, clinical breast examination (CBE), and breast self-examination (BSE), particularly in low-resource settings where diagnostic facilities are limited [6, 7]. Among these, BSE is a quick, simple, cost-effective, non-invasive technique that can be performed by women themselves to detect early breast abnormalities, potentially improving early diagnosis and prognosis [8].

In low- and middle-income countries (LMICs), such as Pakistan, late-stage diagnosis of breast cancer remains prevalent, contributing to increased morbidity and mortality. One of the primary contributing factors is a lack of awareness and education regarding breast cancer and its early signs [9]. For example, many women seek medical attention only after symptoms become pronounced or severe [10], often resulting in diagnoses at advanced stages. Additional challenges include limited access to trained healthcare professionals and inadequate diagnostic and treatment infrastructure, all of which further hinder effective breast cancer control and management in many communities [9].

Unfortunately, Pakistan bears the highest load of breast cancer among Asian countries. In 2022, approximately 19.3 million cancer cases and 10 million cancer-related deaths were reported nationwide [11, 12]. Breast cancer affects approximately one in every nine Pakistani women [13], with an incidence rate of 69 per 100,000 [14], which is significantly higher than the 19 per 100,000 reported in India [13]. Alarmingly, recent epidemiological data indicate a growing number of younger Pakistani women being diagnosed with breast cancer [12]. Community pharmacists are widely accessible across urban and rural areas in Pakistan and are often the first point of contact within the healthcare system. Their accessibility, regular patient interaction, and trusted role in public health interventions position them well to deliver preventive education.

Given these challenges and the utility of available services, there is a critical need for effective awareness strategies to promote early detection behaviours. So, the objective of this study was to determine the impact of pharmacist-led educational services in promoting breast cancer awareness and their influence on the self-directed behaviour of BSE practice.

## Methodology

### Study design and setting

**Fig. 1:**
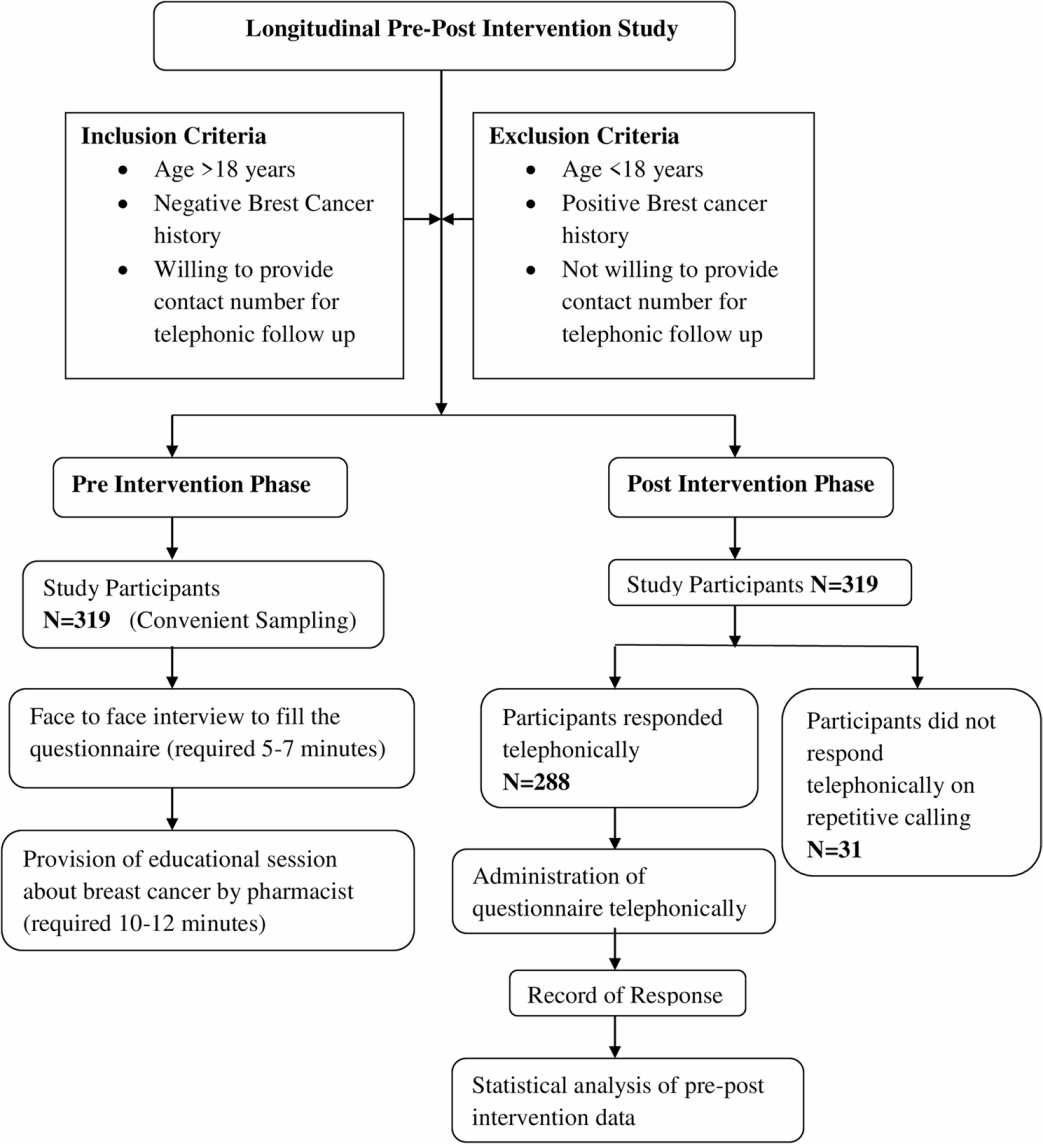
Flow diagram of participants included and interviewed for educational intervention in pre and post-phase of the study

It is a longitudinal pre-post-intervention study conducted using self-designed questionnaires. The study was conducted in pre-selected community pharmacies (*n* = 8) in different areas of Rawalpindi (Rwp) and Islamabad region from November 2021 to May 2022. Walk-in female customers who visited the community pharmacy for general medication needs, aged > 18 years, with no history of breast cancer, were recruited for the study. They must be willing to provide a contact number and sign the written informed consent, which was essential. 319 recruited females were included using a convenient sampling (Fig. 1).

### Questionnaire validation

The questionnaire was validated by experts at the Federal Breast Screening Centre, Pakistan Institute of Medical Sciences (PIMS), Islamabad, Pakistan, prior to its use. The questionnaire was first piloted with 40 participants aged 18 to 60. After minor modifications to the questionnaire, the reliability score (0.78) was calculated based on the pilot study before being used for data collection.

### Data collection forms

The questionnaire was divided into four sections:

1. Personal Information: The data included demographic information (age, marital status, education level, and occupation), as well as age at first childbirth (if applicable), age at menarche, and use of contraceptives (of any type).
2. Knowledge about breast cancer: This section included standard questions that investigated the participants’ knowledge of breast cancer symptoms and risk factors.
3. Knowledge about breast cancer diagnostic techniques: This part assessed the awareness of breast cancer diagnostic techniques (breast self-examination, clinical breast examination, and mammography) and breast self-examination practice.
4. Misconceptions and feedback: This section had questions that could identify any misconception(s) about breast cancer. It also included a feedback question about the educational services provided by the community pharmacists.

### Administration of questionnaire

#### Pre-intervention phase

During the pre-intervention phase, walk-in female visitors at selected pharmacies were asked for permission to participate in a structured data collection by the principal investigator. Following consent, the principal investigator completed the questionnaires as answered by the subjects, followed by an update on education about cancer in accordance with American Cancer Society (ACS) guidelines (Questionnaire attached as a supplementary file). The education session was conducted in a private or semi-private area within the pharmacy, ensuring confidentiality and participant comfort.

The education session included information about breast cancer symptoms, risk factors, diagnostic techniques, mammography guidelines, and screening centers available in Islamabad and Rawalpindi, Pakistan. For better understanding, the participants were also provided with a handout containing information in the local language (Urdu) about the method for BSE and map charting on available screening centers located at PIMS, Pakistan.

#### Post-intervention phase (follow-up)

After a three-month gap, the same women participants were contacted telephonically, blinded from the PI to overcome any possible predisposition, and the post-intervention phase questionnaires were filled out. The same questionnaire about breast cancer awareness was administered to assess whether there had been any changes in women’s self-reported awareness levels and BSE usage (Questionnaire attached as a supplementary file).

### Statistical analysis

The data collected were recorded in SPSS version 25. The percentage frequency and paired samples t-test were calculated for each numerical score/mean of the questionnaire variable. The analysis was done to compare pre- and post-data to evaluate the impact of educational sessions in improving breast cancer awareness and influencing BSE practice behaviour.

## Results

The study was conducted on 319 participants between 18 and 68, who were included in selected community pharmacies in twin cities (Islamabad and Rawalpindi). Baseline demographic characteristics included that 142 (44.5%) of the females visiting the pharmacies were under 30 years old. Among them, 213 (66.7%) of participants were married. In the pre-intervention group, 23 females held a higher university degree, whereas this count declined to 19 in the post-intervention group. Only four women knew their families had a history of breast cancer when asked about it, and 38% didn’t know about any prior history. (Table 1)

**Table 1:** Demographic characteristics of study participants

	Pre		Post	
	*n*	%	*n*	%
Age				
18–28	142	44.5	138	47.9
29–38	98	30.7	83	28.8
39–48	55	17.2	48	16.7
49–58	20	6.3	16	5.6
59–68	4	1.3	3	1.0
Marital status				
Single	103	32.2	101	35.1
Married	213	66.7	184	63.9
Divorced	1	0.3	1	0.7
Widow	2	0.6	2	0.3
Educational status				
No education	9	2.8	4	1.39
Primary education	86	26.9	76	26.4
Secondary education	201	63.0	189	65.6
Tertiary education	23	7.2	19	6.6
Residence				
Rural	286	89.7	271	94.1
Urban	33	10.3	17	5.9
Family history of breast cancer				
Yes	4	1.25	3	10.4
No	193	60.5	211	73.2
Do not know	122	38.2	74	25.7
Total	319		**288**	

### Knowledge about breast cancer symptoms

**Fig. 2:**
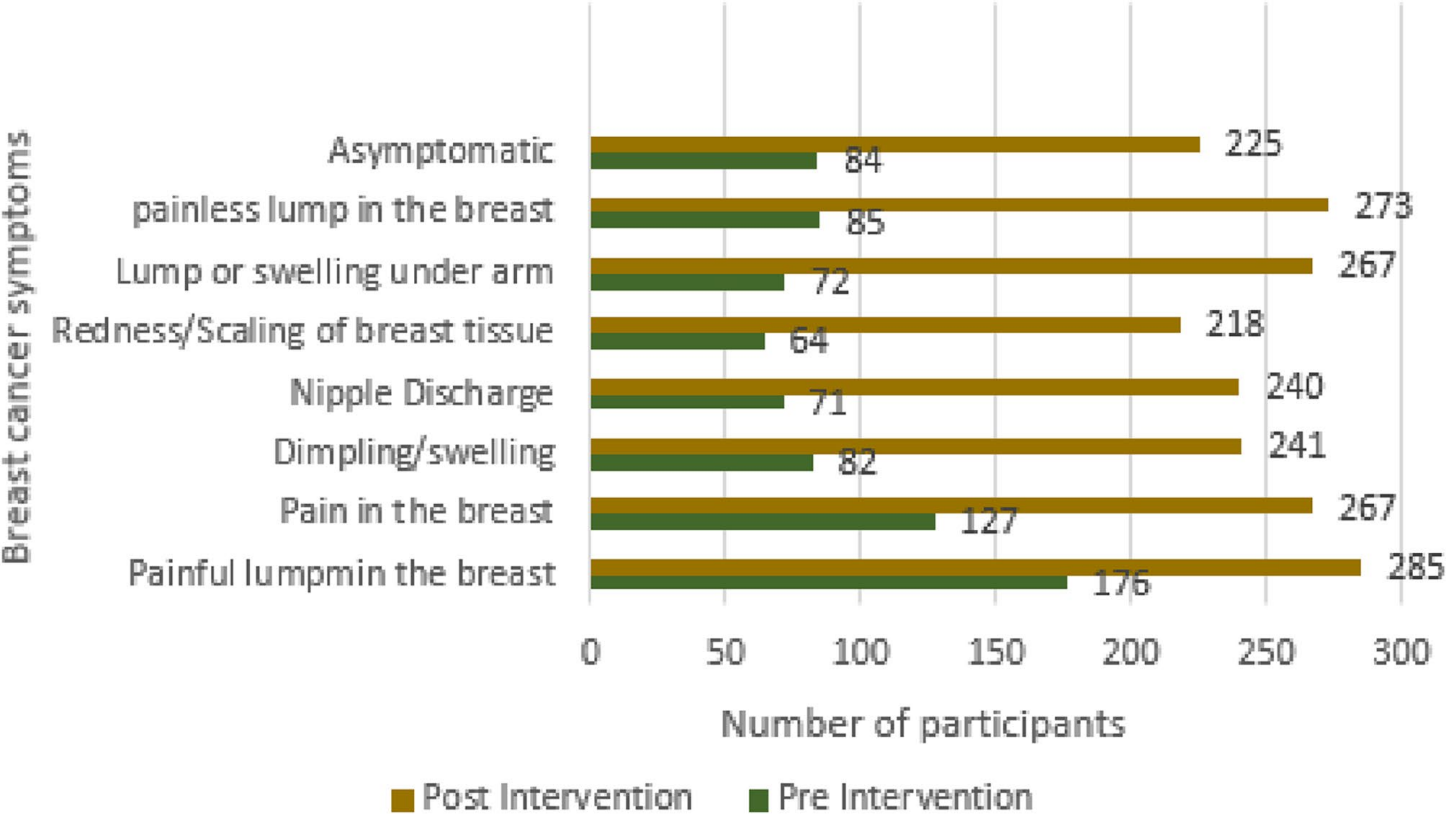
Improved knowledge of study participants on breast cancer symptoms during pre- and post-intervention phases

Early diagnosis of the disease is aided by familiarity with the early symptoms of breast cancer. Through a breast cancer educational intervention, the study participants were made aware of the symptoms of breast cancer. As a result, awareness of the symptoms tends to rise in the post-intervention phase compared to the pre-intervention phase. Examples include an increase in awareness of the symptoms “lump in the breast” (from 55.2% to 99), “pain in the breast” (from 39.8% to 92.7%), “dimpling/swelling” (from 25.7% to 83.7%), “nipple discharge” (from 22.3% to 83.3%), “redness/scaling of breast skin” (from 20.1% to 75.4%), “lump or swelling under arms” (from 22.6% to 92.7%), and “being asymptomatic” (from 26.3% to 78.1%) in pre-post intervention phases, respectively (Fig. 2).

### Knowledge of breast cancer risk factors

**Fig. 3:**
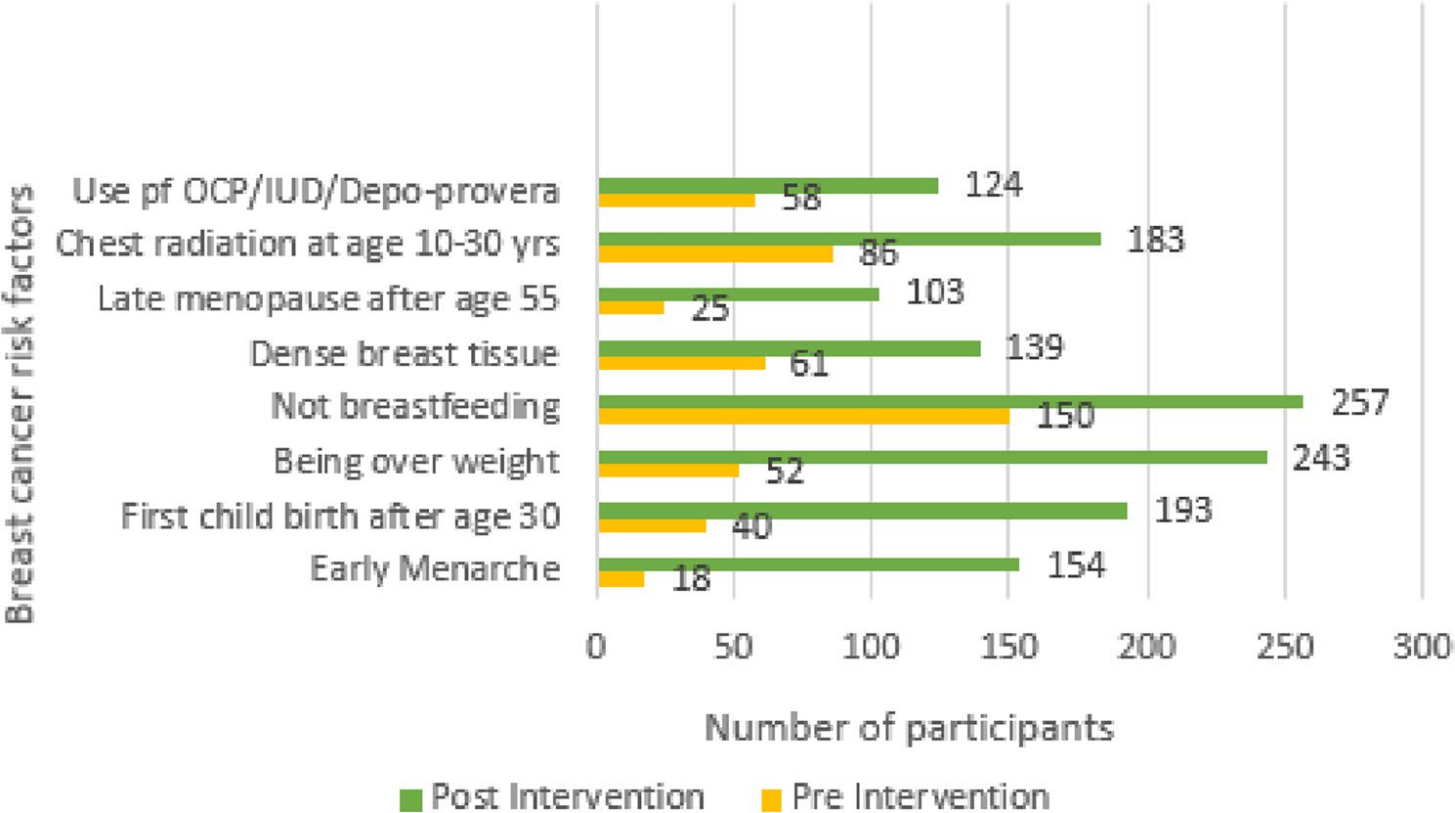
Improved knowledge of breast cancer risk factors following pharmacist-led education during pre and post-intervention phases

Knowledge of breast cancer risk factors that may affect a woman’s chance of getting breast cancer is crucial in raising awareness among women. The study participants were provided with an understanding of the risk factors through an educational intervention session provided by the pharmacist. Overall, the awareness of different risk factors improved after the education session. For example, there was an increase in awareness following education on risk factor “Early menarche” (from 5.6% to 53.5%), “age at first childbirth” (from 12.5% to 67%), being overweight (from 16.3% to 84.4%), “not breastfeeding” (from 47.5% to 89.2%), “have dense breast tissue” (from 19.1% to 48.3%), “late menopause after age 55” (from 7.8% to 35.8%), “have had a chest radiation at age 30 years” (from 27% to 63.5%) and “using of oral contraceptive pills (OCP)/intra-uterine device (IUD)” (from 18.2% to 43.1%) in pre and post-intervention phase, respectively (Fig. 3).

### Awareness of breast cancer diagnostic techniques

**Fig. 4:**
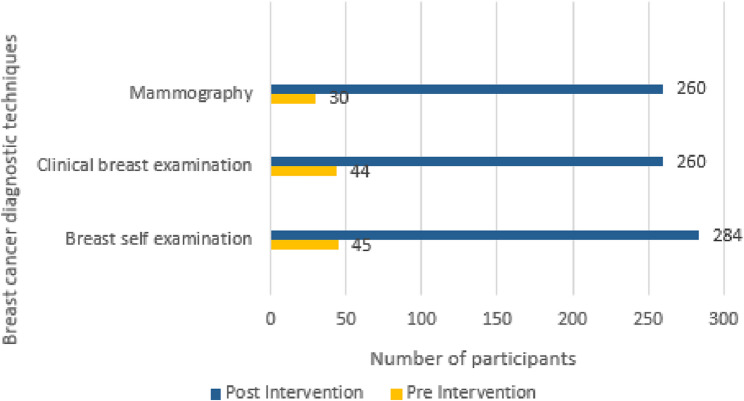
Improved knowledge of breast cancer diagnostics techniques following pharmacist-led education during pre and post-intervention phases

The study participants were also provided knowledge of breast cancer diagnostic techniques through pharmacist-led breast cancer educational sessions. Similarly, there was increased awareness across the board following educational sessions. For example, there was an increase in awareness about the BSE as a breast cancer diagnostic technique (from 14.1% to 98.6%), “CBE awareness” (from 13.8% to 90.3%), and “mammography” (from 9.4% to 90.3%) as a breast cancer diagnostic technique in pre and post-intervention phases, respectively (Fig. 4).

### Evidence of improvement in breast cancer awareness about symptoms, risk factors, and diagnostic techniques

A Paired t-test analysis (Table 2) revealed a significant improvement in breast cancer awareness among the study participants, encompassing a comprehensive understanding of breast cancer symptoms, risk factors, and diagnostic techniques. The statistical assessment demonstrated a *p-value* of 0.000 for all three variables, unequivocally affirming a highly significant association.

**Table 2:** Improvement in awareness levels

Paired Samples		Paired Differences					t-value	Sig.(2- tailed)
		Mean	Std. Deviation	Std. Error Mean	95% Confidence Interval of the Difference			
					Lower	Upper		
Pair 1	**Pre-Symptoms** **Post-Symptoms**	−4.660	2.461	0.145	−4.945	−4.374	−32.129	0.000*
Pair 2	**Pre-Risk factors** **Post-Risk factors**	−3.260	2.470	0.146	−3.547	−2.974	−22.398	0.000*
Pair 3	**Pre-Techniques** **Post-Techniques**	−2.417	0 0.918	0.054	−2.523	−2.310	−44.663	0.000*

*P-value < 0.01 = highly significant

### Improvement in BSE practice behavior

**Fig. 5:**
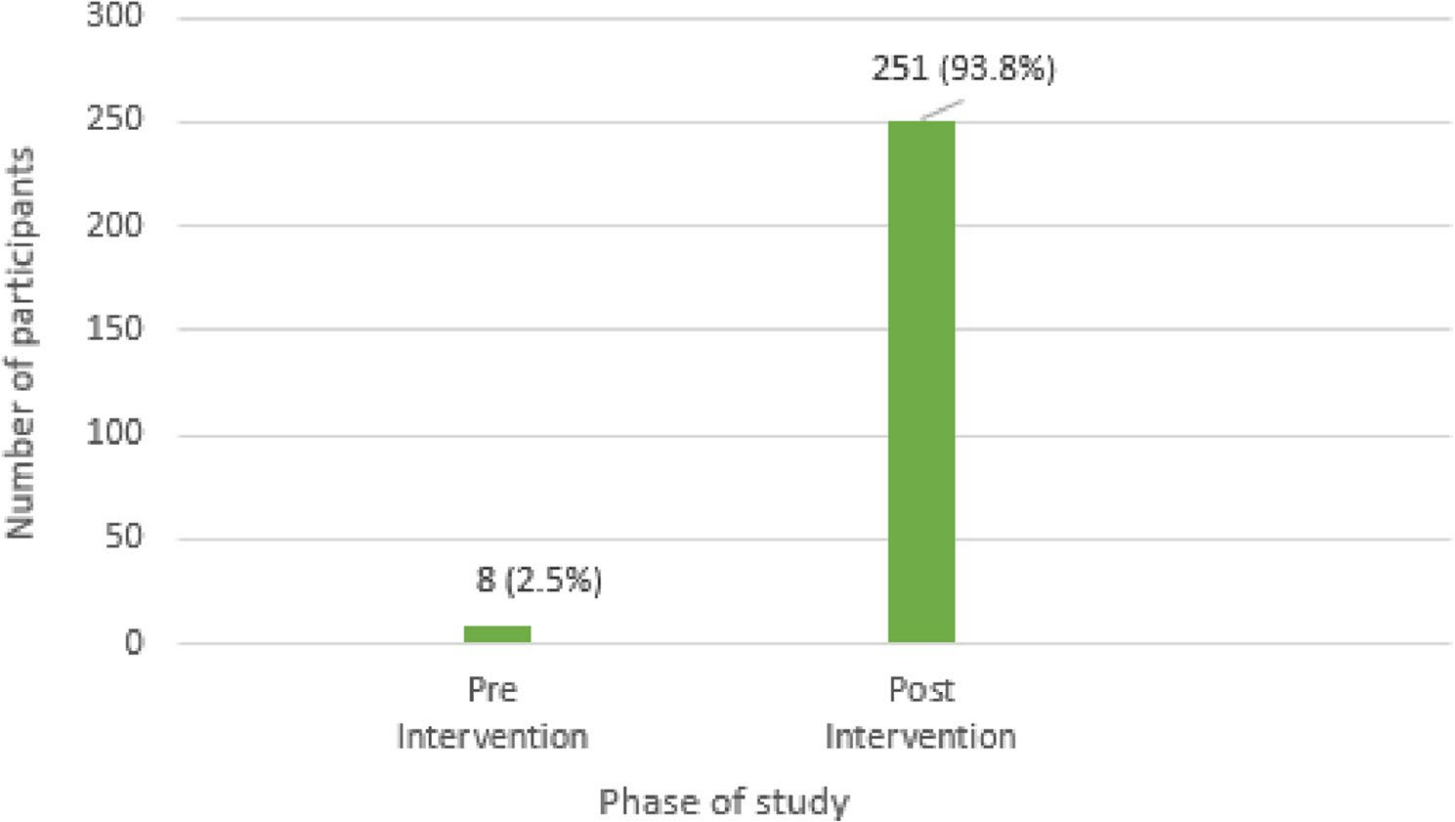
Practice of BSE following pharmacist-led education pre and post-intervention phase

**Fig. 6:**
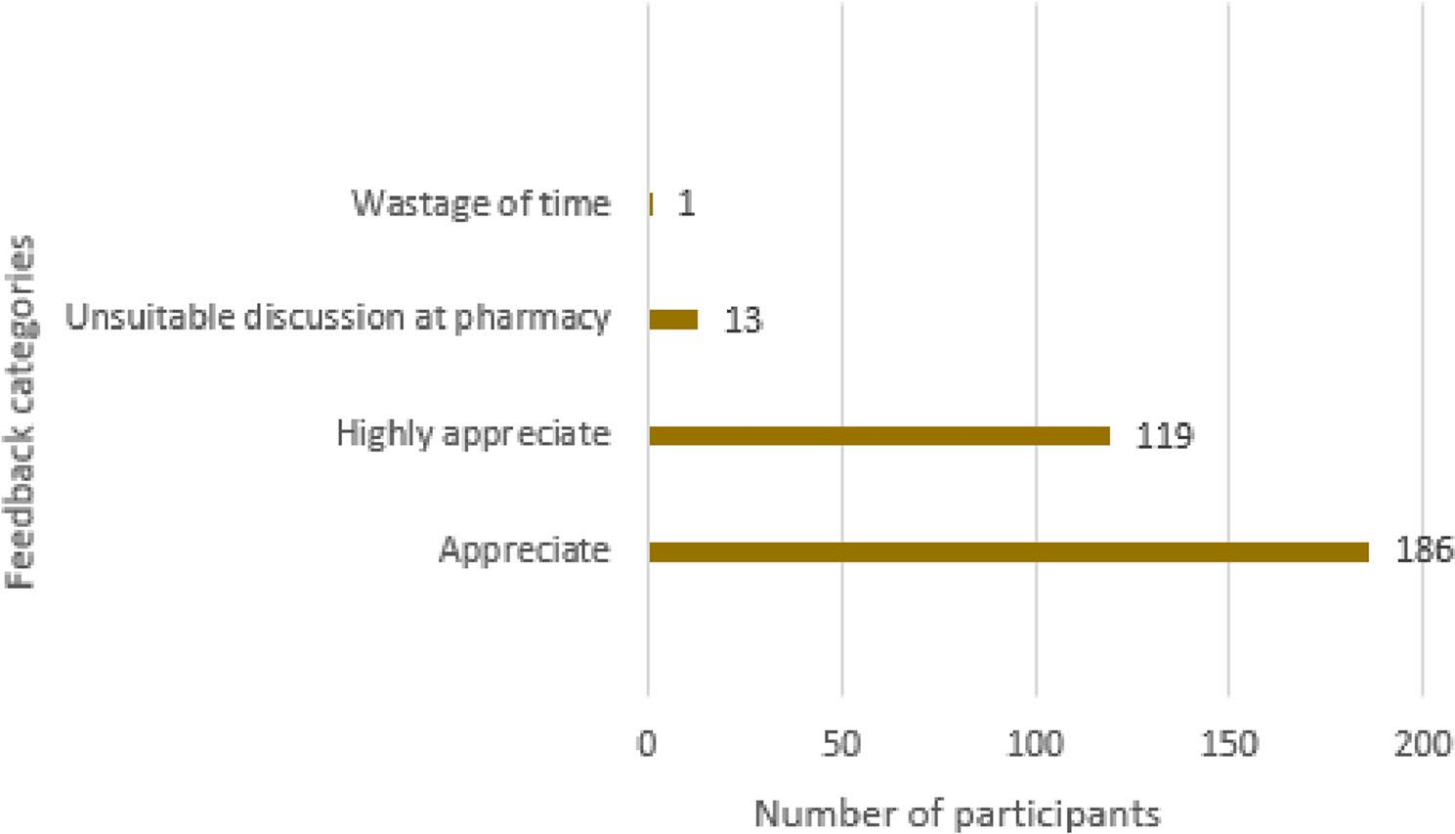
Feedback on pharmacist-led breast cancer educational sessions conducted at the community pharmacies

The proportion of study participants practicing the BSE in the pre-intervention phase was extremely low (2.5%) (Fig. 5). It improved up to 93.8% after the education session post-intervention (Fig. 6), indicating the importance of the educational session.

### Effect on familiarity and practice of mammography

During the pre-phase of the study, it is worth noting that merely three out of the 319 participants had undergone a mammogram. A greater majority (90%) of the study participants were entirely uninformed about the mammography screening procedure. However, following the informative educational session, there was a remarkable increase in awareness regarding mammography, with up to 90% of participants exhibiting improved knowledge in the post-intervention phase. This positive shift in awareness resulted in 16 participants actively seeking mammography screening at the designated center during the post-intervention phase, as reflected in Table 3.

**Table 3:** Awareness of participants about mammography pre- and post-intervention phase

Variable	Category	Pre		Post	
Familiarity and experience of Mammography	Familiar and experienced	**n**	**%**	**n**	**%**
		3	0.9	16	5.6
	Familiar but never experienced	29	9.1	243	84.4
	Not familiar and never experienced	287	90.0	29	10.1
	Total	319		288	

### Feedback on pharmacist-led educational session

In the evaluation of the educational session, study participants were invited to provide feedback. A large number of respondents, precisely 95.6% of the participants, stated their appreciation for the pharmacist-led educational session. Among these respondents, 58.3% expressed a high level of appreciation, while 37.30% responded with a general appreciation. Only a small portion, accounting for 4.07% of participants, found the educational session to be “unsuitable,” and an even smaller portion, comprising 0.3% of respondents, regarded it as a waste of time (Fig. 6).

## Discussion

The present study was designed to evaluate the impact of pharmacist-led educational interventions on breast cancer awareness and BSE practices among women in Pakistan. Our findings demonstrate that a structured, pharmacy-based awareness session significantly improved participants’ knowledge of breast cancer risk factors, signs and symptoms, and available screening options. Furthermore, the intervention enhanced self-reported adoption of BSE practices, highlighting the potential role of community pharmacists as accessible health professionals in promoting early detection strategies, particularly in LMICs where specialized breast cancer services are limited.

Our study, which has incorporated this recommendation, suggests that the educational intervention conducted at community pharmacies is a good foundation and source of knowledge that should be fully utilized to improve breast cancer awareness among the local communities. Increased breast cancer awareness, which results in early cancer confirmation followed by individualized, effective treatment, is highly recommended for breast cancer control in LMICs [6]. Among the Asian countries, the highest occurrence of breast cancer is reported in Pakistan [3, 14]. It is believed that the lack of awareness is one of the major causes of inflated breast cancer mortality [9].

In the current study, we confirmed the severe lack of awareness about breast cancer symptoms, risk factors, and diagnostic techniques, as seen in the pre-intervention phase. The results are consistent with the earlier studies conducted in Pakistan, which identified poor awareness as the major obstacle to improving breast cancer survival [15, 16]. Awareness is therefore a critical factor, as cancer treatments are most effective when the disease is detected at an early stage.

The recommendation of BSE in women above 20 years is essential for individual risk realization and early detection [17], especially in a population with high prevalence, such as Pakistan. The post-intervention phase showed an increase in awareness (from 19.1% to 98.3%) and practice of BSE following pharmacist-led education. The recent finding has significantly improved the understanding and practice of BSE. The finding aligns with a community-based study conducted in Pakistan, where educational content-based intervention improved awareness about BSE [7, 18]. Considering the low BSE help for screening breast cancer, awareness about BSE may potentially be beneficial in LMICs [9].

Additionally, the correct timing of performing BSE is crucial when trying to detect the early symptoms of breast cancer. Based on the ACS guidelines, the best time to perform BSE is a week following menstruation. Only 4.7% of study participants were aware of this knowledge during the pre-intervention phase, which improved considerably to 93.8% in the intervention phase. Similarly, the number of study participants practicing BSE improved post-intervention as well. The current intervention study helped improve study participants’ confidence in BSE practice, and approximately 56.3% of study participants reported feeling confident about detecting early symptoms of breast cancer by performing BSE. The finding is consistent with the results of a study conducted in Ethiopia, where the practical competency of BSE increased from 10 (16.4%) to 43 (70.5%) satisfactory levels after the delivery of a teaching intervention [19].

The present study is the first to report that a pharmacist-led educational intervention can improve BSE knowledge and practice, which has previously been confirmed as an effective way to identify early symptoms and improve survival chances [6, 17]. The pharmacist-led educational session delivers the required information that CBE is one of the screening techniques for early breast cancer detection, as recommended by the ACS for females aged > 40 years or older. Unfortunately, only 4% of the study participants were familiar with the recommended age for mammography during the pre-intervention phase, which improved up to 86% in participants during the post-intervention phase. About 90% of the study participants became familiar with mammography as a BSE in the post-intervention phase, indicating that the pharmacist-led education improved.

In our study, the total number of study participants greater than age 40 was 46. In the pre-intervention phase, only 3 (6.52%) participants (of age > 40) had gone through mammography, but the percentage improved to 34.7 during the post-intervention phase. The improvement in screening behaviour may be attributed to more study participants becoming familiar with educational intervention through mammography, a safe method employed for early detection to improve survival chances, which improved the courage for mammography exposure. According to the findings of a study, the key factors that hinder women from undergoing mammography include being shy, fear of death/losing breast or being diagnosed with cancer, considering mammography harmful, and transportation difficulties [20]. Considering all the barrier perceptions, the participants were also informed about the safety and availability of free screening centers in the twin cities of Islamabad & Rawalpindi.

Approximately 6.89% of participants knew free screening centres exist in Islamabad and Rawalpindi. The educational services about breast cancer offered by the pharmacist at the pharmacy provided the study participants with complete information about free screening centers and their locations. This information was beneficial to promoting early mammography screening behavior in a country like Pakistan, where economic constraints and lack of resources also restrict women from undergoing expensive screening for breast cancer.

Our findings confirmed that there is limited awareness (43.6%) of breast cancer risk factors. For example, many participants have the misconception that wearing tight bras may contribute to breast cancer development. Educational intervention in the post-intervention phase analysis rectified the participants’ misconceptions. The positive feedback of study participants depicts the social acceptance of pharmacist-led educational services at community pharmacies. 95% of participants appreciated the educational service and the distribution of breast cancer educational pamphlets. The pharmacist can expand the expectations of their services for the patients and generate opportunities to contribute to health promotion activities [21].

Pharmacists’ engagement in health promotion to elevate awareness levels about health-related issues and preventive methods is a persistent recommendation of the WHO [22, 23]. Community pharmacists are the healthcare providers who are widely approachable and reliable. The findings of the present study of improved breast cancer awareness resulting from educational sessions conducted by the pharmacist support the evidence and agree that the pharmacist can potentially contribute to breast cancer control. The findings of this study prospectively suggest that the concerned health authorities make effective use of the underutilized counseling skills of pharmacists to combat the monster of breast cancer.

### Limitations of the study

This study has several limitations. The absence of a control group and reliance on self-reported data may introduce bias, including social desirability and recall bias. Outcomes were assessed only in the short term, so this method is prone to social desirability bias and may have led participants to over-report BSE behavior following the educational session. Although no adverse effects such as anxiety or misinterpretation of normal breast changes were observed, these were not formally measured. The educational content was intentionally simplified to match the literacy level of participants, which may have limited the inclusion of complex but important topics such as genetic risk factors. Lastly, while the principal investigator received formal training, a lack of standardized pharmacist training across multiple sites may limit the generalizability and scalability of the intervention.

## Conclusion

The study showed a significant improvement in breast cancer awareness and BSE practice using the pharmacist-led educational intervention. Raising awareness in younger women is especially important in Pakistan, where cultural barriers, stigma, and lack of education often prevent timely health-seeking behaviors. Educating women at a younger age may encourage earlier presentation and improve future health literacy. By utilizing the pharmacists at community pharmacies, comprehensive strategies could be introduced to escalate public breast cancer awareness nationally.

## Supplementary Information

Supplementary Material 1.

Supplementary Material 2.

Supplementary Material 3.

Supplementary Material 4.

## Data Availability

All data generated or analyzed during this study are included in this article.
