# Supplementary material for: Impact of pharmacist-led educational services in promoting breast cancer awareness
Source: BMC Womens Health. 2025 Sep 29;25:461. doi: 10.1186/s12905-025-04035-0 (PMC12481808; doi:10.1186/s12905-025-04035-0)
Supplement: Supplementary file 1 — Supplementary Material 1. [file 12905_2025_4035_MOESM1_ESM.docx]

**Breast Cancer Awareness Assessment**

**Pre-Intervention Phase Questionnaire**

**Instructions:**

Listen each statement carefully. Choose one of the options for each statement indicating how well that statement describes you.

***Personal Information:* Name *__________________***

**Contact # ___________________**

***Name of Pharmacy Visited__________________________ Date_________***

1. **What was your age at last birthday? (In years) _____________________**
2. **What is your weight? _____________________**
3. **What is your marital status?**

| Single | Married | Divorced | Widow |
| --- | --- | --- | --- |

1. **What was your age at first childbirth, If any? _____________________**
2. **Do you use any of these?**

| Birth Control Pills | IUD | Depo provera | None |
| --- | --- | --- | --- |

1. **What was your age at menarche? _____________________**
2. **What is your Education?**

| Primary | Secondary | Higher. Secondary | Graduate | Medical Education | Illiterate |
| --- | --- | --- | --- | --- | --- |

1. **What is your occupation?**

| Teaching | Health Field | Office Staff | Housewife | Student | Other |
| --- | --- | --- | --- | --- | --- |

1. **Where do you live?**

| Islamabad | Rawalpindi | Other |
| --- | --- | --- |

❶Knowledge about Breast Cancer

**1)Do you Know the breast cancer disease?**

| a | Have heard the name only |
| --- | --- |
| b | Well known |
| c | don’t know about the disease |

**2)What are the symptoms of breast cancer?**

| a | painful lump in breast |
| --- | --- |
| b | pain in the breast |
| c | Dimpling/swelling |
| d | Nipple discharge (other than breast Milk) |
| e | Redness/scaling of the breast skin |
| f | Lump or swelling under arm |
| g | painless lump in breast |
| h | Asymptomatic |

**3)What could be the risk factor for breast cancer?**

| a | Early Menarche |
| --- | --- |
| b | first child after age 30 |
| c | being overweight |
| d | Not breast feeding |
| e | Dense Breast Tissue |
| f | late menopause after age 55 |
| g | Chest radiation in age 10-30 yrs |
| h | Use of OCP/IUD/ Depo-provera |

**4)From which source you got the information about breast cancer?**

| a | Health Care Provider |
| --- | --- |
| b | TV/Radio/Seminar |
| c | Internet/YouTube |
| d | Mother/Friend/Relative |
| e | None |

**5)Do you have any family history of breast cancer?**

| a | Yes |
| --- | --- |
| b | No |

**6)If yes then what was your relation with the diagnosed person?**

| a | Mother/Sister |
| --- | --- |
| b | Aunty/Cousin |
| c | Grand Mother |
| d | Other |
| e | None |

**❷Knowledge about Diagnostic Techniques of Breast Cancer**

**7)What technique(s) used for breast cancer Diagnosis?**

| a | Breast Self-Examination |
| --- | --- |
| b | Clinical Breast Examination |
| c | Mammography |
|  |  |

**8)Do you know how to perform breast self-examination? (BSE)**

| a | Yes |
| --- | --- |
| b | No |

**9)Who taught you the procedure of breast self-examination?**

| a | Mother/Sister |
| --- | --- |
| b | Teacher |
| c | Health care provider |
| d | Internet/youtube |
| e | None |

**10)What should be the frequency of breast self-examination?**

| A | Daily |
| --- | --- |
| B | Once a week |
| C | Once in a month |
| D | Once in a year |
| E | Don’t know |

**11)What is the best to perform breast self-examination?**

| a | During Menstruation |
| --- | --- |
| b | Right After menstruation |
| c | Week later Menstruation |
| d | in pregnancy |
| e | During breastfeeding |
| f | Don’t know |

**12)How often do you practice breast self-examination?**

| a) Weekly | b) Monthly |
| --- | --- |
| c) Occasionally | d) Never practiced |

1. **Do you feel confident that you can identify any lump or abnormality through Breast Self-Examination after BSE education by pharmacist?**

| a) Yes | b) No |
| --- | --- |

**14)Have you ever had Clinical Breast Examination?**

| a) Yes | b) No |
| --- | --- |

**15)What should be the frequency of Clinical Breast Examination?**

| a) Within Three months | b) Once in a year |
| --- | --- |
| c) After childbirth | d) When observe any lump |
| e) Don’t know |  |

**16)Do you have any personal experience of breast Mammography?**

| a) Yes, Familiar | b) No but familiar |
| --- | --- |
| c) No and not familiar |  |

**17)What should be the age to start Mammography?**

| a) after 18-25 years | b) After 30-35 years |
| --- | --- |
| c) after 40-50 years | d) after 55-60 years |
| e) No idea |  |

**❸Assessment of Misconception about Breast Cancer/Survival chances /Screening Facilities**

**18)Are you familiar with free breast cancer screening clinics available in Twin cities?**

| a) Yes | b) No |
| --- | --- |
| c)Don’t exist any |  |

**19)What do we think that Breast Cancer is ……………?**

| a) a communicable disease | b) attacks only married females |
| --- | --- |
| c)Always result in breast loss | d)of no cure |
| e) Caused by tight Bra | f) attack female with family history |

**20)Do you think a breast cancer female can lead a normal life after treatment?**

| a) Yes | b) No |
| --- | --- |
| c)Death is obvious |  |

**21)What is your opinion about the breast cancer educational session at pharmacy?**

| a) I appreciate | b) I highly appreciate |
| --- | --- |
| c) unsuitable discussion at pharmacy | d) wastage of time |
