# Supplementary material for: Impact of pharmacist-led educational services in promoting breast cancer awareness
Source: BMC Womens Health. 2025 Sep 29;25:461. doi: 10.1186/s12905-025-04035-0 (PMC12481808; doi:10.1186/s12905-025-04035-0)
Supplement: Supplementary file 3 — Supplementary Material 3. [file 12905_2025_4035_MOESM3_ESM.pdf]

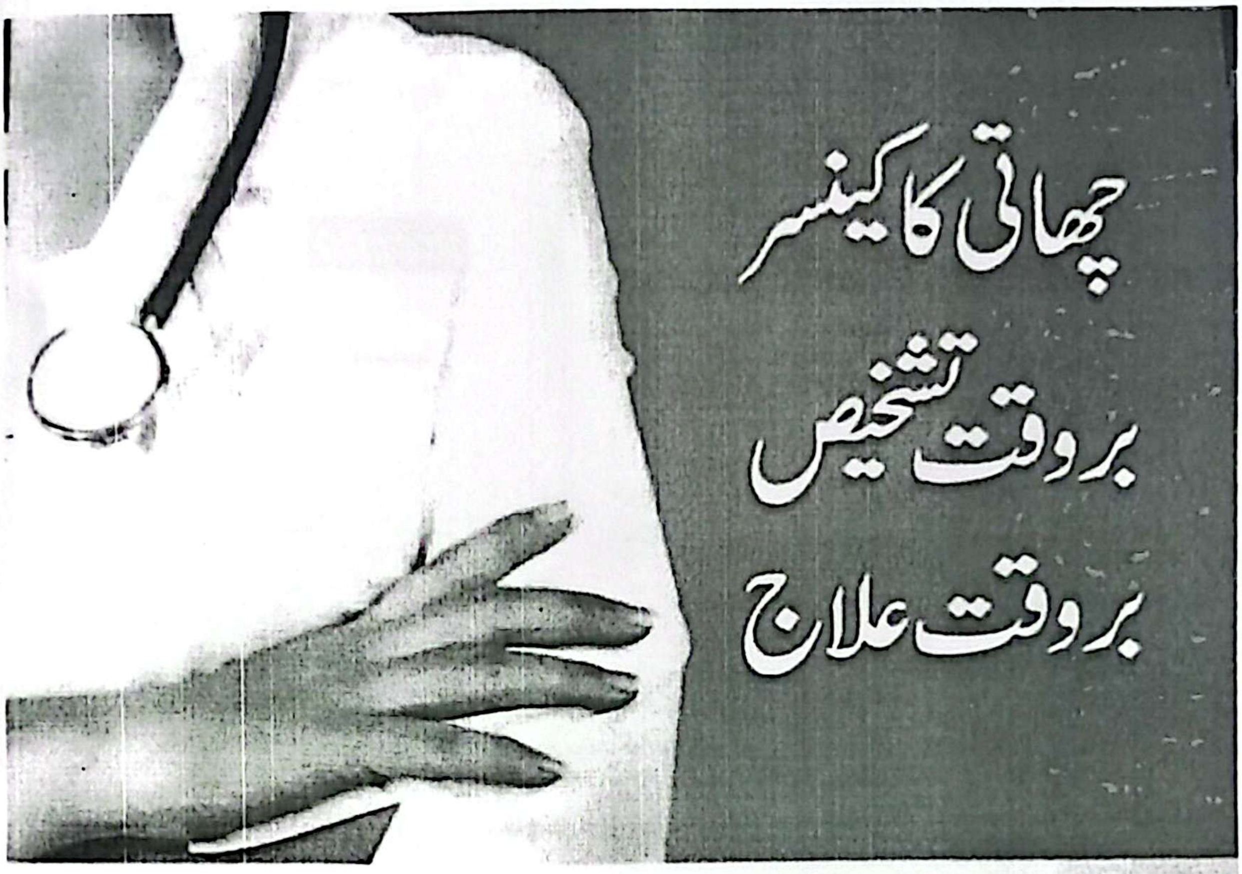

پاکستان میں خواتین کو ہونے والے کینسر میں سب سے زیادہ شرح

چھاتی کے کینسر کی ہے۔

آپ کی صحت آپکی اولین ترجیح ہے۔

تین بہت آسان لیکن موثر اقدام آپ کو چھاتی کے کینسر سے بچا سکتے ہیں۔

1 ماہانہ خود معائنہ
2 میموگراف (چھاتی کا ایکسرے)
3 چھاتیوں کا پیشہ ورانہ معائنہ

Federal Breast Cancer Screening Centre
•AWARENESS •DETECTION •CURE

CamScanner

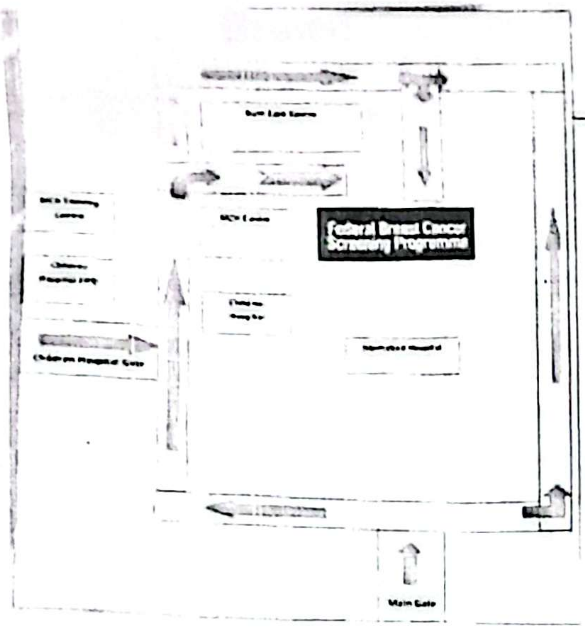

1 چھاتیوں کا معائنہ خود کیسے کریں؟

اس معائنے میں ایک آئینے اور آپ کی درمیانی انگلیوں کی ضرورت ہے۔

دیکھنا کیا ہے؟

- جلد کی ساخت اور رنگ میں کوئی تبدیلی مثلاً سرخی وغیرہ
- نپل سے خون ملی رطوبت کا اخراج ہونا
- جلد یا نپل کا اندر کی جانب دھنس جانا
- کوئی گٹھلی جو صاف نظر آتی ہو
- جلد میں گڑھا

نہاتے ہوئے معائنہ کرنا:

نہاتے ہوئے اپنی چھاتیوں کا معائنہ کریں۔ گیلی جلد پر معائنہ بآسانی ہو جاتا ہے۔ انگلیوں کو سیدھا رکھیں اور انگوٹھا استعمال نہ کریں۔ دائیں ہاتھ سے بائیں چھاتی اور بائیں ہاتھ سے دائیں چھاتی کا معائنہ کریں۔ اطمینان کر لیں کہ کہیں کوئی گانٹھ، ابھار یا سختی تو محسوس نہیں ہوتی۔

سیدھے لیٹ کر معائنہ کرنا:

دائیں چھاتی کے معائنے کیلئے دائیں کندھے کے نیچے تکیہ یا تولیہ تہہ کر کے رکھ لیں اور دایاں ہاتھ سر کے نیچے رکھیں۔ اس طرح چھاتی کے عضلات سینے پر ہموار سے پھیل جاتے ہیں۔ بائیں ہاتھ کی انگلیوں کو سیدھا رکھیں اور دباتے ہوئے چھاتی کا دائرے کی طرح مکمل معائنہ کریں۔ اپنی چھاتی کو ایک گھڑی فرض کر لیں۔ معائنہ بارہ کے ہندسے سے شروع کریں۔ ایک بار آئیں، دوپہر پہنچیں اور اسی طرح دائرہ مکمل کر کے بارہ پر واپس آ جائیں۔ چھاتی کی نچلی گولائی کے ساتھ ساتھ ایک مضبوط عضلاتی ابھار ایک عام بات ہے۔ آخر میں نپل کو انگلی اور انگوٹھے کے درمیان دبائیں۔ اس میں سے خون یا کوئی رطوبت نکلے تو فوراً ڈاکٹر سے رجوع کریں۔ ایسی گانٹھ جو دو چار ہفتے تک چھوٹی نہ ہو فوری طور پر ڈاکٹر کو دکھانی چاہئے۔

سینے کے معائنے کا بہتر وقت:

مہینے میں ایک بار ماہواری سے ایک ہفتے بعد اپنائیے کیونکہ ان دنوں چھاتیاں نہ تو زیادہ حساس ہوتی ہیں نہ ہی زیادہ پھولی ہوئی ہوتی ہیں۔ ماہواری بند ہونے کی صورت میں مہینے کے پہلے دن معائنہ اپنا معمول بنالیں۔ Hysterectomy ہو چکی ہو تو اپنے ڈاکٹر سے معلوم کریں کہ آپ کیلئے چھاتیوں کے معائنے کا بہتر دن کونسا ہے۔

اگر آپ کو چھاتی میں کوئی گٹھلی محسوس ہو یا گرہ یا سختی محسوس ہو تو آپ کو کیا کرنا چاہئے:

اگر معائنے کے دوران کوئی گٹھلی یا گرہ محسوس ہو یا کسی قسم کا کوئی مواد نکلے تو فوراً ڈاکٹر سے رجوع کریں اور خائف نہ ہوں کیونکہ گٹھلی کینسر کے علاوہ بھی ہو سکتی ہے۔

2 میموگراف یا چھاتی کا ایکسرے

اس میں جو ایکسرے ہوتا ہے اس سے بہت چھوٹے کینسر کا بھی پتہ چل جاتا ہے جو ہاتھ سے محسوس نہیں ہوتا۔ یہ چھاتی کے کینسر سے محفوظ رہنے کا ایک اہم طریقہ ہے۔ 35 سے 40 سال کی عمر میں ایک Baseline ایکسرے ہونا چاہئے۔ 40 سے 49 سال کی عمر میں ہر سال نہیں تو ہر دوسرے سال یہ ایکسرے ہونا چاہئے۔

3 چھاتیوں کا پیشہ ورانہ معائنہ

چھاتیوں کا پیشہ ورانہ معائنہ کسی ایسے شخص سے کرایا جا سکتا ہے جس نے اس کی تربیت حاصل کی ہو۔ یہ سال میں ایک بار کرایا جاتا ہے جس کے ذریعے ابتدائی تبدیلی بھی معلوم کی جا سکتی ہے جو شاید آپ نظر انداز کر دیں۔

Federal Breast Cancer Screening Centre
AWARENESS • DETECTION • CURE

Behind MCH indoor and adjacent to Burn Care Centre PIMS, Hospital, Islamabad, Ph: 051-9107712, 051-9107713
Main Radiology Office, Ph: 051-9107671
