# Supplementary material for: Impact of pharmacist-led educational services in promoting breast cancer awareness
Source: BMC Womens Health. 2025 Sep 29;25:461. doi: 10.1186/s12905-025-04035-0 (PMC12481808; doi:10.1186/s12905-025-04035-0)
Supplement: Supplementary file 4 — Supplementary Material 4. [file 12905_2025_4035_MOESM4_ESM.pdf]

Breast Cancer Signs and Symptoms

Knowing how your breasts normally look and feel is an important part of breast health. Finding breast cancer as early as possible gives you a better chance of successful treatment. But knowing what to look for does not take the place of having regular mammograms (/cancer/breast-cancer/screening-tests-and-early-detection/mammograms.html) and other screening tests (/cancer/breast-cancer/screening-tests-and-early-detection.html). Screening tests can help find breast cancer in its early stages, before any symptoms appear.

The most common symptom of breast cancer is a new lump or mass. A painless, hard mass that has irregular edges is more likely to be cancer, but breast cancers can be tender, soft, or rounded. They can even be painful. For this reason, it is important to have any new breast mass, lump, or breast change checked by a health care professional experienced in diagnosing breast diseases.

Other possible symptoms of breast cancer include:

- Swelling of all or part of a breast (even if no distinct lump is felt)
- Skin irritation or dimpling (sometimes looking like an orange peel)
- Breast or nipple pain
- Nipple retraction (turning inward)
- Redness, scaliness, or thickening of the nipple or breast skin
- Nipple discharge (other than breast milk)

Sometimes a breast cancer can spread to lymph nodes under the arm or around the collar bone and cause a lump or swelling there, even before the original tumor in the breast is large enough to be felt. Swollen lymph nodes should also be checked by a health care provider.

Although any of these symptoms can be caused by things other than breast cancer, if you have them, they should be reported to a health care professional so that the cause can be found.

Because mammograms do not find every breast cancer, it is important for you to be aware of changes in your breasts and to know the signs and symptoms of breast cancer.

Written by

CamScanner

Lifestyle-related Breast Cancer Risk Factors

A risk factor is anything that affects your chance of getting a disease, such as breast cancer. But having a risk factor, or even many, does not mean that you are sure to get the disease.

Certain breast cancer risk factors are related to personal behaviors, such as diet and exercise. Other lifestyle-related risk factors include decisions about having children and taking medicines that contain hormones.

Drinking alcohol

Drinking alcohol (/cancer/cancer-causes/diet-physical-activity/alcohol-use-and-cancer.html) is clearly linked to an increased risk of breast cancer. The risk increases with the amount of alcohol consumed. Compared with non-drinkers, women who have 1 alcoholic drink a day have a very small increase in risk. Those who have 2 to 3 drinks a day have about a 20% higher risk compared to women who don't drink alcohol. Excessive alcohol consumption is known to increase the risk of other cancers, too.

The American Cancer Society recommends that women who drink have no more than 1 drink a day.

Being overweight or obese

Being overweight or obese (/cancer/cancer-causes/diet-physical-activity/body-weight-and-cancer-risk.html) after menopause increases breast cancer risk. Before menopause your ovaries make most of your estrogen, and fat tissue makes only a small amount. After menopause (when the ovaries stop making estrogen), most of a woman's estrogen comes from fat tissue. Having more fat tissue after menopause can raise estrogen levels and increase your chance of getting breast cancer. Also, women who are overweight tend to have higher blood insulin levels. Higher insulin levels have been linked to some cancers, including breast cancer.

Still, the link between weight and breast cancer risk is complex. For instance, risk appears to be increased for women who gained weight as an adult, but may not be increased among those who have been overweight since childhood. Also, excess fat in the waist area may affect risk more than the same amount of fat in the hips and thighs. Researchers believe that fat cells in various parts of the body have subtle differences that may explain this.

CamScanner

weight might also have different effects on different types of breast cancer. For example, some research suggests that being overweight before menopause might increase your risk of triple-negative breast cancer.

The American Cancer Society recommends you stay at a healthy weight throughout your life and avoid excess weight gain by balancing your food intake with physical activity.

Not being physically active

Evidence is growing that regular physical activity reduces breast cancer risk, especially in women past menopause. The main question is how much activity is needed. Some studies have found that even as little as a couple of hours a week might be helpful , although more seems to be better.

Exactly how physical activity might reduce breast cancer risk isn't clear, but it may be due to its effects on body weight, inflammation, hormones, and energy balance.

The American Cancer Society recommends (/healthy/eat-healthy-get-active/acs-guidelines-nutrition-physical-activity-cancer-prevention.html) that adults get at least 150 minutes of moderate intensity or 75 minutes of vigorous intensity activity each week (or a combination of these), preferably spread throughout the week.

Not having children

Women who have not had children or who had their first child after age 30 have a slightly higher breast cancer risk overall. Having many pregnancies and becoming pregnant at an early age reduces breast cancer risk. Still, the effect of pregnancy seems to be different for different types of breast cancer. For a certain type of breast cancer known as triple-negative, pregnancy seems to increase risk.

Not breastfeeding

Some studies suggest that breastfeeding may slightly lower breast cancer risk, especially if it's continued for 1½ to 2 years. But this has been hard to study, especially in countries like the United States, where breastfeeding for this long is uncommon.

The explanation for this possible effect may be that breastfeeding reduces a woman's total number of lifetime menstrual cycles (the same as starting menstrual periods at a later age or going through early menopause).

Birth control

Some birth control methods use hormones, which might increase breast cancer risk.

CamScanner

oral contraceptives: Most studies have found that women using oral contraceptives (birth control pills) have a slightly higher risk of breast cancer than women who have never used them. Once the pills are stopped, this risk seems to go back to normal over time. Women who stopped using oral contraceptives more than 10 years ago do not appear to have any increased breast cancer risk.

Birth control shot: Depo-Provera is an injectable form of progesterone that's given once every 3 months for birth control. Some studies have found that women currently using birth-control shots seem to have an increase in breast cancer risk, but it appears that there is no increased risk in women 5 years after they stop getting the shots.

Birth control implants, intrauterine devices (IUDs), skin patches, vaginal rings: These forms of birth control also use hormones, which in theory could fuel breast cancer growth. Some studies have shown a link between use of hormone-releasing IUDs and breast cancer risk, but few studies have looked at the use of birth control implants, patches, and rings and breast cancer risk.

When thinking about using hormonal birth control, women should discuss their other risk factors for breast cancer with their health care provider.

Hormone therapy after menopause

Hormone therapy (/cancer/cancer-causes/medical-treatments.html) with estrogen (often combined with progesterone) has been used for many years to help relieve symptoms of menopause and help prevent osteoporosis (thinning of the bones). This treatment goes by many names, such as *post-menopausal hormone therapy* (PHT), *hormone replacement therapy* (HRT), and *menopausal hormone therapy* (MHT).

There are 2 main types of hormone therapy. For women who still have a uterus (womb), doctors generally prescribe estrogen and progesterone (known as *combined hormone therapy* or HT). Progesterone is needed because estrogen alone can increase the risk of cancer of the uterus. For women who've had a hysterectomy (who no longer have a uterus), estrogen alone can be used. This is known as *estrogen replacement therapy* (ERT) or just *estrogen therapy (ET)*.

Combined hormone therapy (HT): Use of combined hormone therapy after menopause increases the risk of breast cancer. It may also increase the chances of dying from breast cancer. This increase in risk can be seen with as little as 2 years of use. Combined HT also increases the likelihood that the cancer may be found at a more advanced stage.

The increased risk from combined HT appears to apply only to current and recent users. A woman's breast cancer risk seems to return to that of the general population within 5 years of stopping treatment.

Bioidentical hormone therapy: The word *bioidentical* is sometimes used to describe versions of estrogen and progesterone with the same chemical structure as those found naturally in people. The use of these hormones has been marketed as a safe way to treat the symptoms of menopause. But because there aren't many studies comparing "bioidentical" or "natural" hormones to synthetic versions of hormones, there's

CamScanner

American Cancer Society

Breast Cancer Risk Factors You Cannot Change

A risk factor is anything that affects your chance of getting a disease, such as breast cancer. But having a risk factor, or even many, does not mean that you are sure to get the disease.

Some risk factors for breast cancer are things you cannot change, such as being a woman, getting older, and having certain gene changes. These make your risk of breast cancer higher.

Being a woman

Simply being a woman is the main risk factor for breast cancer. Men can get breast cancer, too, but this disease is about 100 times more common in women than in men.

Getting older

As you get older, your risk of breast cancer goes up. Most breast cancers are found in women age 55 and older.

Certain inherited genes

About 5% to 10% of breast cancer cases are thought to be hereditary, meaning that they result directly rom gene defects (called *mutations*) passed on from a parent.

***BRCA1* and *BRCA2*:** The most common cause of hereditary breast cancer is an inherited mutation in the *BRCA1* or *BRCA2* gene. In normal cells, these genes help make proteins that repair damaged DNA. Mutated ersions of these genes can lead to abnormal cell growth, which can lead to cancer.

- If you have inherited a mutated copy of either gene from a parent, you have a higher risk of breast cancer.
- On average, a woman with a *BRCA1* or *BRCA2* gene mutation has about a 7 in 10 chance of getting breast cancer by age 80. This risk is also affected by how many other family members have had breast cancer. (It goes up if more family members are affected.)

CamScanner

- Women with one of these mutations are more likely to be diagnosed with breast cancer at a younger age, as well as to have cancer in both breasts. They also have a higher risk of developing some other cancers, mainly ovarian cancer.
- In the United States, *BRCA* mutations are more common in Jewish people of Ashkenazi (Eastern Europe) origin than in other racial and ethnic groups, but anyone can have them.

Changes in other genes: Other gene mutations can also lead to inherited breast cancers. These gene mutations are much less common, and most of them do not increase the risk of breast cancer as much as the *BRCA* genes.

- ***ATM*:** The *ATM* gene normally helps repair damaged DNA (or helps kill the cell if the damaged can't be fixed). Inheriting 2 abnormal copies of this gene causes the disease *ataxia-telangiectasia*. Inheriting one abnormal copy of this gene has been linked to a high rate of breast cancer in some families.
- ***TP53*:** The *TP53* gene gives instructions for making a protein called *p53* that helps stop the growth of abnormal cells. Inherited mutations of this gene cause *Li-Fraumeni syndrome*. People with this syndrome have an increased risk of breast cancer, as well as some other cancers such as leukemia, brain tumors, and sarcomas (cancers of bones or connective tissue). This mutation is a rare cause of breast cancer.
- ***CHEK2*:** The *CHEK2* gene is another gene that normally helps with DNA repair. A *CHEK2* mutation can increase breast cancer risk about 2-fold.
- ***PTEN*:** The *PTEN* gene normally helps regulate cell growth. Inherited mutations in this gene can cause *Cowden syndrome*, a rare disorder that puts people at higher risk for both non-cancer and cancer tumors in the breasts, as well as growths in the digestive tract, thyroid, uterus, and ovaries.
- ***CDH1*:** Inherited mutations in this gene cause *hereditary diffuse gastric cancer*, a syndrome in which people develop a rare type of stomach cancer. Women with mutations in this gene also have an increased risk of invasive lobular breast cancer.
- ***STK11*:** Defects in this gene can lead to *Peutz-Jeghers syndrome*. People affected with this disorder have pigmented spots on their lips and in their mouths, polyps (abnormal growths) in the urinary and digestive tracts, and a higher risk of many types of cancer, including breast cancer.
- ***PALB2*:** The *PALB2* gene makes a protein that interacts with the protein made by the *BRCA2* gene. Mutations in this gene can lead to a higher risk of breast cancer.

Mutations in several other genes have also been linked to breast cancer, but these account for only a small number of cases.

Genetic testing: Genetic testing can be done to look for mutations in the *BRCA1* and *BRCA2* genes (or less commonly in other genes such as *PTEN* or *TP53*). While testing can be helpful in some cases, not every woman needs to be tested, and the pros and cons need to be considered carefully.

One concern is that some genetic tests are promoted to doctors and the public without giving full information. For example, a test for a small number of BRCA1 and BRCA2 gene mutations (/cancer/breast-cancer/understanding-a-breast-cancer-diagnosis/breast-cancer-her2-status.html) has been approved by

CamScanner

the FDA. However, there are more than 1,000 known BRCA mutations, and the ones included in the approved test are not the most common ones. This means there are many BRCA mutations that would not be detected by this test.

If you're thinking about genetic testing, it's strongly recommended that you first talk to a genetic counselor, nurse, or doctor who can explain these tests. It's very important to understand what genetic testing can and can't tell you, and to carefully weigh the benefits and risks of genetic testing before these tests are done. Testing costs a lot and might not be covered by some health insurance plans.

Our section on genetics and cancer (/cancer/cancer-causes/genetics.html) has more information about genetic mutations and testing for them.

Having a family history of breast cancer

It's important to note that most women (about 8 out of 10) who get breast cancer *do not* have a family history of the disease. But women who have close blood relatives with breast cancer have a higher risk:

- Having a first-degree relative (mother, sister, or daughter) with breast cancer almost doubles a woman's risk. Having 2 first-degree relatives increases her risk about 3-fold.
- Women with a father or brother who have had breast cancer also have a higher risk of breast cancer.

Overall, less than 15% of women with breast cancer have a family member with this disease.

Having a personal history of breast cancer

A woman with cancer in one breast has a higher risk of developing a new cancer in the other breast or in another part of the same breast. (This is different from a recurrence or return of the first cancer.) Although this risk is low overall, it's even higher for younger women with breast cancer.

Your race and ethnicity

Overall, white women are slightly more likely to develop breast cancer than African-American women. But in women under age 45, breast cancer is more common in African-American women. African-American women are also more likely to die from breast cancer at any age. Asian, Hispanic, and Native American women have a lower risk of developing and dying from breast cancer.

Having dense breast tissue

CamScanner

Breasts are made up of fatty tissue, fibrous tissue, and glandular tissue. Someone is said to have dense breasts (on a mammogram) when they have more glandular and fibrous tissue and less fatty tissue. Women with dense breasts on mammogram have a risk of breast cancer that is about 1.5 to 2 times that of women with average breast density. Unfortunately, dense breast tissue can also make it harder to see cancers on mammograms.

A number of factors can affect breast density, such as age, menopausal status, the use of certain drugs (including menopausal hormone therapy), pregnancy, and genetics.

For more information, see our information on breast density and mammograms (/cancer/breast-cancer/screening-tests-and-early-detection/mammograms/breast-density-and-your-mammogram-report.html).

Certain benign breast conditions

Women diagnosed with certain benign (non-cancer) breast conditions may have a higher risk of breast cancer. Some of these conditions are more closely linked to breast cancer risk than others. Doctors often divide benign breast conditions into 3 groups, depending on how they affect this risk.

Non-proliferative lesions: These conditions don't seem to affect breast cancer risk, or if they do, the increase in risk is very small. They include:

- Fibrosis and/or simple cysts (sometimes called *fibrocystic changes* or *disease*)
- Mild hyperplasia
- Adenosis (non-sclerosing)
- Phyllodes tumor (benign)
- A single papilloma
- Fat necrosis
- Duct ectasia
- Periductal fibrosis
- Squamous and apocrine metaplasia
- Epithelial-related calcifications
- Other tumors (lipoma, hamartoma, hemangioma, neurofibroma, adenomyoepithelioma)

Mastitis (infection of the breast) is not a tumor and does not increase the risk of breast cancer.

Proliferative lesions without atypia (cell abnormalities): In these conditions there's excessive growth of cells in the ducts or lobules of the breast, but the cells don't look very abnormal. These conditions seem to raise a woman's risk of breast cancer slightly. They include:

CamScanner

- Usual ductal hyperplasia (without atypia)
- Fibroadenoma
- Sclerosing adenosis
- Several papillomas (called *papillomatosis*)
- Radial scar

Proliferative lesions with atypia: In these conditions, the cells in the ducts or lobules of the breast tissue grow excessively, and some of them no longer look normal. These types of lesions include:

- Atypical ductal hyperplasia (ADH) (/treatment/understanding-your-diagnosis/tests/understanding-your-pathology-report/breast-pathology/atypical-hyperplasia.html)
- Atypical lobular hyperplasia (ALH) (/treatment/understanding-your-diagnosis/tests/understanding-your-pathology-report/breast-pathology/atypical-hyperplasia.html)

Breast cancer risk is about 4 to 5 times higher than normal in women with these changes. If a woman also has a family history of breast cancer and either hyperplasia or atypical hyperplasia, she has an even higher risk of breast cancer.

For more information, see Non-cancerous Breast Conditions (/cancer/breast-cancer/non-cancerous-breast-conditions.html).

Lobular carcinoma in situ (LCIS)

In LCIS (/treatment/understanding-your-diagnosis/tests/understanding-your-pathology-report/breast-pathology/lobular-carcinoma-in-situ.html), cells that look like cancer cells are growing in the lobules of the milk-producing glands of the breast, but they are not growing through the wall of the lobules. LCIS is also called *lobular neoplasia*. It's sometimes grouped with ductal carcinoma in situ (DCIS) (/cancer/breast-cancer/understanding-a-breast-cancer-diagnosis/types-of-breast-cancer/dcis.html) as a non-invasive breast cancer, but it differs from DCIS in that it doesn't seem to become invasive cancer if it isn't treated.

Women with LCIS have a much higher risk of developing cancer in either breast.

Starting menstruation (periods) early

Women who have had more menstrual cycles because they started menstruating early (especially before age 12) have a slightly higher risk of breast cancer. The increase in risk may be due to a longer lifetime exposure to the hormones estrogen and progesterone.

Going through menopause after age 55

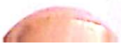

CamScanner

Women who have had more menstrual cycles because they went through menopause later (after age 55) have a slightly higher risk of breast cancer. The increase in risk may be because they have a longer lifetime exposure to the hormones estrogen and progesterone.

Having radiation to your chest

Women who were treated with radiation therapy (/treatment/treatments-and-side-effects/treatment-types/radiation.html) to the chest for another cancer (such as Hodgkin disease or non-Hodgkin lymphoma) when they were younger have a significantly higher risk for breast cancer. This varies with the patient's age when they got radiation. The risk is highest if you had radiation as a teen or young adult, when your breasts were still developing. Radiation treatment after age 40 does not seem to increase breast cancer risk.

Exposure to diethylstilbestrol (DES)

From the 1940s through the early 1970s some pregnant women were given an estrogen-like drug called DES because it was thought to lower their chances of losing the baby (miscarriage). These women have a slightly increased risk of developing breast cancer. Women whose mothers took DES during pregnancy may also have a slightly higher risk of breast cancer.

To learn more, see our information about DES exposure (/cancer/cancer-causes/medical-treatments/des-exposure.html).

Written by References

American Cancer Society

The American Cancer Society medical and editorial content team (/cancer/acs-medical-content-and-news-staff.html)

Our team is made up of doctors and oncology certified nurses with deep knowledge of cancer care as well as journalists, editors, and translators with extensive experience in medical writing.

ast Medical Review: September 6, 2017 | Last Revised: September 6, 2017

merican Cancer Society medical information is copyrighted material. For reprint requests, please see our ontent Usage Policy (/about-us/policies/content-usage.html).

MORE IN BREAST CANCER

CS CamScanner

American Cancer Society Recommendations for the Early Detection of Breast Cancer

Finding breast cancer early and getting state-of-the-art cancer treatment are the most important strategies to prevent deaths from breast cancer. Breast cancer that's found early, when it's small and has not spread, is easier to treat successfully. Getting regular screening tests is the most reliable way to find breast cancer early. The American Cancer Society has screening guidelines for women at average risk of breast cancer, and for those at high risk for breast cancer.

What are screening tests?

The goal of screening tests for breast cancer is to find it before it causes symptoms (like a lump that can be felt). **Screening** refers to tests and exams used to find a disease in people who don't have any symptoms. **Early detection** means finding and diagnosing a disease earlier than if you'd waited for symptoms to start.

Breast cancers found during screening exams are more likely to be smaller and still confined to the breast. The size of a breast cancer and how far it has spread are some of the most important factors in predicting the **prognosis** (outlook) of a woman with this disease.

American Cancer Society screenings recommendations for women at average breast cancer risk

These guidelines are for women at **average risk** for breast cancer. For screening purposes, a woman is considered to be at average risk if she doesn't have a personal history of breast cancer, a strong family history of breast cancer, or a genetic mutation known to increase risk of breast cancer (such as in a *BRCA* gene), and has not had chest radiation therapy before the age of 30. (See below for guidelines for women at high risk.)

Women between 40 and 44 have the option to start screening with a mammogram every year.

CamScanner

Women 45 to 54 should get mammograms every year.

Women 55 and older can switch to a mammogram every other year, or they can choose to continue yearly mammograms. Screening should continue as long as a woman is in good health and is expected to live 10 more years or longer.

All women should understand what to expect when getting a mammogram for breast cancer screening – what the test can and cannot do.

Mammograms

Regular mammograms (/cancer/breast-cancer/screening-tests-and-early-detection/mammograms.html) can help find breast cancer at an early stage, when treatment is most successful. A mammogram can find breast changes that could be cancer years before physical symptoms develop. Results from many decades of research clearly show that women who have regular mammograms are more likely to have breast cancer found early, are less likely to need aggressive treatment like surgery to remove the breast (mastectomy) and chemotherapy, and are more likely to be cured.

Mammograms are not perfect. They miss some cancers. And sometimes a woman will need more tests to find out if something found on a mammogram is or is not cancer. There's also a small possibility of being diagnosed with a cancer that never would have caused any problems had it not been found during screening. It's important that women getting mammograms know what to expect and understand the benefits and limitations of screening.

Clinical breast exam and breast self-exam

Research has not shown a clear benefit of regular physical breast exams done by either a health professional (clinical breast exams) or by yourself (breast self-exams). There is very little evidence that these tests help find breast cancer early when women also get screening mammograms. Most often when breast cancer is detected because of symptoms (such as a lump), a woman discovers the symptom during usual activities such as bathing or dressing. **Women should be familiar with how their breasts normally look and feel and report any changes to a health care provider right away.**

American Cancer Society screening recommendations for women at high risk

Women who are at **high risk** for breast cancer based on certain factors should get an MRI (/cancer/breast-cancer/screening-tests-and-early-detection/breast-mri-scans.html) and a mammogram every year, typically starting at age 30. This includes women who:

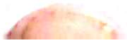

CamScanner

- Have a lifetime risk of breast cancer of about 20% to 25% or greater, according to risk assessment tools that are based mainly on family history (see below)
- Have a known *BRCA1* or *BRCA2* gene mutation (/cancer/breast-cancer/risk-and-prevention/breast-cancer-risk-factors-you-cannot-change.html) (based on having had genetic testing)
- Have a first-degree relative (parent, brother, sister, or child) with a *BRCA1* or *BRCA2* gene mutation, and have not had genetic testing themselves
- Had radiation therapy to the chest when they were between the ages of 10 and 30 years
- Have Li-Fraumeni syndrome, Cowden syndrome, or Bannayan-Riley-Ruvalcaba syndrome, or have first-degree relatives with one of these syndromes

The American Cancer Society recommends against MRI screening for women whose lifetime risk of breast cancer is less than 15%.

There's not enough evidence to make a recommendation for or against yearly MRI screening for women who have a higher lifetime risk based on certain factors , such as:

- Having a personal history of breast cancer, ductal carcinoma in situ (DCIS) (/cancer/breast-cancer/understanding-a-breast-cancer-diagnosis/types-of-breast-cancer/dcis.html), lobular carcinoma in situ (LCIS) (/cancer/breast-cancer/non-cancerous-breast-conditions/lobular-carcinoma-in-situ.html), atypical ductal hyperplasia (ADH), or atypical lobular hyperplasia (ALH) (/cancer/breast-cancer/non-cancerous-breast-conditions/hyperplasia-of-the-breast-ductal-or-lobular.html)
- Having "extremely" or "heterogeneously" dense breasts (/cancer/breast-cancer/screening-tests-and-early-detection/mammograms/breast-density-and-your-mammogram-report.html) as seen on a mammogram

If MRI is used, it should be in addition to, not instead of, a screening mammogram. This is because although an MRI is more likely to detect cancer than a mammogram, it may still miss some cancers that a mammogram would detect.

Most women at high risk should begin screening with MRI and mammograms when they are 30 and continue for as long as they are in good health. But a woman at high risk should make the decision to start with her health care providers, taking into account her personal circumstances and preferences.

Tools used to assess breast cancer risk

Several risk assessment tools are available to help health professionals estimate a woman's breast cancer risk. These tools give approximate, rather than precise, estimates of breast cancer risk based on different combinations of risk factors and different data sets.

Because the different tools use different factors to estimate risk, they may give different risk estimates for the same woman. Two models could easily give different estimates for the same person.

CamScanner

American Cancer Society

Disproven or Controversial Breast Cancer Risk Factors

There are many factors that research has shown are not linked to breast cancer. You may see information online or hear about these disproven or controversial risk factors, but it's important to learn the facts.

Antiperspirants

nternet and e-mail rumors have suggested that chemicals in underarm antiperspirants are absorbed hrough the skin, interfere with lymph circulation, and cause toxins to build up in the breast, eventually eading to breast cancer.

Based on the available evidence (including what we know about how the body works), there is little if any eason to believe that antiperspirants increase the risk of breast cancer. For more information, see Antiperspirants and Breast Cancer Risk (/cancer/cancer-causes/antiperspirants-and-breast-cancer-isk.html).

Bras

nternet and e-mail rumors and at least one book have suggested that bras cause breast cancer by obstructing lymph flow. There is no good scientific or clinical basis for this claim, and a 2014 study of more than 1,500 women found no association between wearing a bra and breast cancer risk.

Induced abortion

Several studies have provided very strong data that neither induced abortions nor spontaneous abortions miscarriages) have an overall effect on the risk of breast cancer. For more detailed information, see Abortion and Breast Cancer Risk (/cancer/cancer-causes/medical-treatments/abortion-and-breast-cancer-risk.html).

Written by   References

CS CamScanner
